# Efficient Light Harvesting and Water Retention Realized by Soybean Protein‐Based Microgels Embedded in Hybrid Sodium Alginate Hydrogels Containing Photocatalyst for Hydrogen Evolution

**DOI:** 10.1002/advs.202505118

**Published:** 2025-11-07

**Authors:** Jie Yu, Neng Hu, Weijia Wang, Lin Lei, Huiqing Fan, Peter Müller‐Buschbaum, Qi Zhong

**Affiliations:** ^1^ Key Laboratory of Intelligent Textile and Flexible Interconnection of Zhejiang Province & Key Laboratory of Advanced Textile Materials & Manufacturing Technology Ministry of Education Zhejiang Sci‐Tech University 928 Second Avenue Hangzhou 310018 China; ^2^ State Key Laboratory of Solidification Processing School of Materials Science and Engineering Northwestern Polytechnical University Xi'an 710072 China; ^3^ Technical University of Munich TUM School of Natural Sciences Department of Physics Chair for Functional Materials James‐Franck‐Str. 1 85748 Garching Germany

**Keywords:** hybrid hydrogels, light harvesting, photocatalytic hydrogen evolution, soybean protein, water retention

## Abstract

The hydrogen evolution and water retention are enhanced by introducing soybean protein‐based microgels into the hybrid sodium alginate (SA) hydrogels containing photocatalyst g‐C_3_N_4_/Pt nanosheets. The soybean protein‐based microgels are prepared from a mixture of soybean protein nanofibers (SPN) and SA. Due to the different refractive indices of SPN and SA, multi‐scattering of incident light on the transparent SPN/SA microgels significantly improves the light‐harvesting capability. The hydrogen evolution rate (HER) significantly rises to 4830 µmol h^−1^ g^−1^, 57% higher than that without SPN/SA microgels. In addition, the ─COOH and ─NH_2_ groups in soybean protein also trigger the formation of additional hydrogen bonding and electrostatic interaction. It prominently reduces the water evaporation rate. After exposure to infrared illumination for 3 h, the weight loss of hybrid SA hydrogels embedded with SPN/SA microgels (mass ratio of SPN to SA = 2:1) is only 5.7%, which is 27% slower than that without SPN/SA microgels. Thus, the embedded SPN/SA microgels not only improve the light‐harvesting but also prolong the lifetime of the hybrid SA hydrogels. They are very suitable for hydrogen production in areas rich in sunlight but poor in water, such as prairies and deserts.

## Graphical abstract

By incorporating SPN/SA microgels into SA hydrogels containing g‐C_3_N_4_/Pt nanosheets, highly efficient light absorption and superior water retention are achieved, thereby significantly enhancing hydrogen evolution efficiency. This is because microgels act as light scattering centers, enabling effective scattering of incident light and improving light trapping capability. Furthermore, the SPN enhances electrostatic interactions and hydrogen bonding with water molecules, leading to improved water retention.

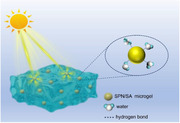

## 1 Introduction

Graphitic carbon nitride (g‐C_3_N_4_) is a metal‐free photocatalyst that can be obtained from a variety of sources and prepared via a relatively simple approach.^[^ ^1^, ^2^, ^3^, ^4^ ^]^ The traditional photocatalysts, such as titanium dioxide (TiO_2_), silver chloride (AgCl), and 2‐(2H‐benzoxniazol‐2‐yl)‐4,6‐bis(1‐methyl‐1‐1‐phenylethyl)phenol (UV‐234), can only absorb UV irradiation, whereas g‐C_3_N_4_ has an advantageous band gap energy of 2.7 eV. Thus, it can absorb visible light energy and carry out photocatalytic reactions, which significantly improves photocatalytic efficiency. Besides that, g‐C_3_N_4_ also exhibits good thermal, chemical, and photochemical stability.^[^ ^5^, ^6^, ^7^, ^8^, ^9^, ^10^, ^11^ ^]^ Moreover, g‐C_3_N_4_ can be combined with large bandgap semiconductors to create heterojunction composites, which facilitate charge separation, expand the surface area, and enhance light absorption.^[^ ^12^, ^13^, ^14^ ^]^ These advantages render g‐C_3_N_4_ a promising metal‐free photocatalyst well‐suited for addressing energy crises and environmental concerns.

However, the typical g‐C_3_N_4_ is in the form of powder. Therefore, it is easy to aggregate and significantly lower the photocatalytic efficiency. To overcome this drawback, nowadays, hydrogels are introduced into the photocatalytic system. In general, hydrogels are defined as a 3D polymer network containing a substantial proportion of water molecules.^[^ ^15^ ^]^ Due to the porous structure and excellent water absorption capability, hydrogels can be used to encapsulate g‐C_3_N_4_ for photocatalytic water splitting.^[^ ^16^, ^17^, ^18^ ^]^ When exposed to solar irradiation, the embedded photocatalysts can effectively split the water molecules into hydrogen. The porous structure and huge amount of water molecules in the network enable the continuous operation of photocatalysis, which is crucial for attaining a stable and sustainable hydrogen evolution.^[^ ^19^, ^20^, ^21^, ^22^, ^23^ ^]^ Moreover, the 3D network effectively separates the g‐C_3_N_4_ powder, which is also favorable for photocatalytic water splitting.^[^ ^24^, ^25^, ^26^ ^]^ As a natural polymer material, sodium alginate (SA) is available from a diverse range of raw materials at a relatively low cost.^[^ ^27^, ^28^, ^29^, ^30^, ^31^ ^]^ SA‐based hydrogels are environmentally friendly and recyclable. Simultaneously, the pre‐adsorption and dynamic adsorption of water molecules on the SA network enable the hydrogels to be effectively hydrated during photocatalytic water splitting, which further enhances the efficiency of hydrogen evolution.

Although the hybrid hydrogels containing g‐C_3_N_4_ solve the aggregation issue of g‐C_3_N_4_ and significantly improve the hydrogen evolution efficiency, they still suffer from poor light harvesting and fast water evaporation when exposed to light illumination. Recently, our group installed PVDF membranes on a hybrid hydrogel surface to reduce the water evaporation rate during the photocatalytic water splitting.^[^ ^26^ ^]^ Although the water retention capability improves, the additional membranes also block the incident light and lower the photocatalytic hydrogen evolution. Thus, it is still a challenge to simultaneously realize efficient light harvesting and low water evaporation for the hybrid hydrogels containing g‐C_3_N_4_. Soybean protein isolate (SPI) is a yellow powder that is readily soluble in water and presents an ideal amino acid structure.^[^ ^32^, ^33^, ^34^, ^35^, ^36^, ^37^, ^38^ ^]^ Due to the existence of ─COOH and ─NH_2_ groups, SPI can form a huge number of hydrogen bonds with water molecules, which can reduce the water evaporation rate. However, the yellow color of SPI will also affect the light‐harvesting capability of the hybrid hydrogels. In addition, SPI cannot easily form gels.^[^ ^39^, ^40^, ^41^, ^42^, ^43^ ^]^ To overcome these drawbacks, SPI is first transferred into the form of nanofibers via heating at high temperatures and shearing at low strength. The obtained soybean protein nanofibers (SPN) are a white powder and become more transparent when dissolved in water.^[^ ^44^, ^45^, ^46^, ^47^, ^48^ ^]^ By further mixing with SA, the obtained SPN/SA can form microgels. Based on the different refractive indices of SPN/SA microgels and hybrid SA hydrogels containing g‐C_3_N_4_, the microgels can act as scattering centers for the incident light. The multi‐scattered visible light in the hybrid hydrogels prominently enhances light‐harvesting. Combining the good water retention ability, the obtained hybrid hydrogels embedded with SPN/SA microgels are very suitable for photocatalytic water splitting.

Based on the above discussion, in our present investigation, SPN/SA microgels are first prepared and introduced into the hybrid SA hydrogel system to simultaneously enhance the light harvesting and water retention capability. The morphology of the obtained SPN/SA microgels, as well as the hybrid SA hydrogels embedded with SPN/SA microgels, are first characterized. The improvement of light absorption via the introduction of the SPN/SA microgels is probed by UV–vis spectroscopy. The hydrogen evolution rate, lifetime, and cyclic performance are measured as well to confirm the excellent performance of the present hybrid hydrogels embedded with SPN/SA microgels.

## 2 Results and Discussion

### 2.1 Morphology of Soybean Protein Nanofibers

**Figure 1:**
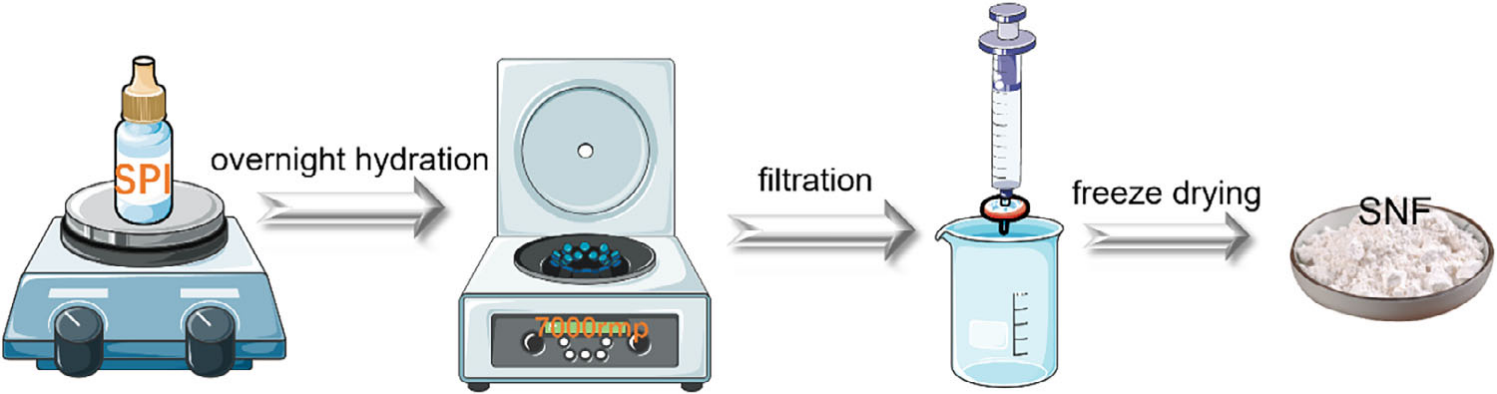
Schematic presentation of the preparation of SPN.

The scheme for the preparation of soy protein nanofibers (SPN) is shown in **Figure** **1**. The differences between soybean isolate protein (SPI) and SPN can be elucidated via transmission electron microscopy (TEM). As presented in Figure S1 (Supporting Information), SPI exhibits a loose aggregation structure in the absence of specific treatment. However, through the application of specific pH conditions and heat treatment, SPI can switch to SPN. The obtained SPN forms a worm‐like structure and possesses a high aspect ratio and random orientation. Neither is commonly observed in natural proteins.

There are prominent differences in the FTIR spectra of SPI and SPN (Figure S2, Supporting Information), which can be attributed to their distinct structure and morphologies. The infrared spectrum of SPI shows the typical features of the protein, mainly including the amide I and II bands. They are visible in the range of 1600–1700 cm^−1^ and 1500–1600 cm^−1^, respectively. Whereas, in the FTIR spectrum of SPN, the amide I band is much weaker. This behavior is due to the fact that during the formation of nanofibers, the soybean protein experiences a partial denaturation. It alters its secondary structure, including an increase in β‐folded content. The formation of nanofibers results in the exposure of amino (3300–3500 cm^−1^) and carboxyl groups (1400–1450 cm^−1^) on the surface, which enhances the characteristic peaks of the corresponding functional groups in the FTIR spectrum. The enhanced O─H stretching vibration peaks (3200–3600 cm^−1^) indicate stronger hydrogen bonding in SPN.

As demonstrated in Figure S3 (Supporting Information), the SPI exhibits a low fluorescence intensity during the measurements. The reason can be attributed to the domination of the α‐helical form in the untreated SPI. Because the ThT fluorescence is strongly correlated to the β‐folded structure, the relatively low amount of β‐folded structure in SPI induces the low intensity. On the contrary, an increased ThT fluorescence intensity is observed in SPN, demonstrating a higher amount of β‐folded structure in SPN.

### 2.2 Morphology of Hybrid SA Hydrogels Embedded with SPN/SA Microgels

**Figure 2:**
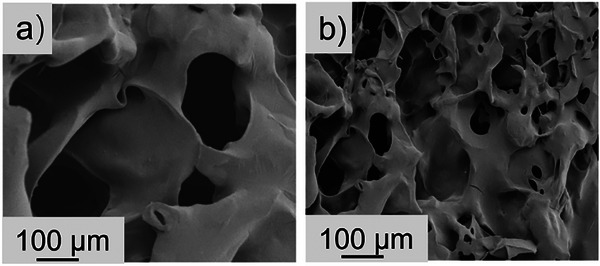
Cross‐section of a) the hybrid SA hydrogels and b) the hybrid SA hydrogels embedded with SPN_2_/SA_1_ microgels.

The morphology of the hybrid SA hydrogels and the hybrid SA hydrogels embedded with SPN_2_/SA_1_ microgels (mass ratio of SPN to SA = 2:1) is probed by SEM. As shown in **Figure** **2**, the cross‐section of the hybrid SA hydrogels presents a porous structure, which is related to two factors. The first one is the physical cross‐linking points in the hydrogels. Instead of covalent bonds, the Ca^2+^ ions form the physical cross‐linking points with the carboxyl groups in the hybrid SA hydrogels. It induces an inhomogeneous structure in the hydrogels and forms a porous structure. The second factor is the existence of g‐C_3_N_4_ nanosheets, which favors the formation of the porous structure.^[^ ^25^ ^]^ Similar to the hybrid SA hydrogels, the cross‐section of the hybrid SA hydrogels embedded with SPN_2_/SA_1_ microgels also shows a porous structure. However, the pores are much smaller. This behavior can be explained by the synergic effect of electrostatic interaction and hydrogen bonding in the hybrid hydrogels after the introduction of SPN. Due to the existence of ─COOH and ─NH_2_ groups in SPN_2_/SA_1_ microgels, both the electrostatic interaction and hydrogen bonding between microgels and hybrid SA hydrogels are enhanced. It not only improves the mechanical properties and structural stability of the hydrogels, but also causes a denser network and a smaller pore size in the hydrogels.

**Figure 3:**
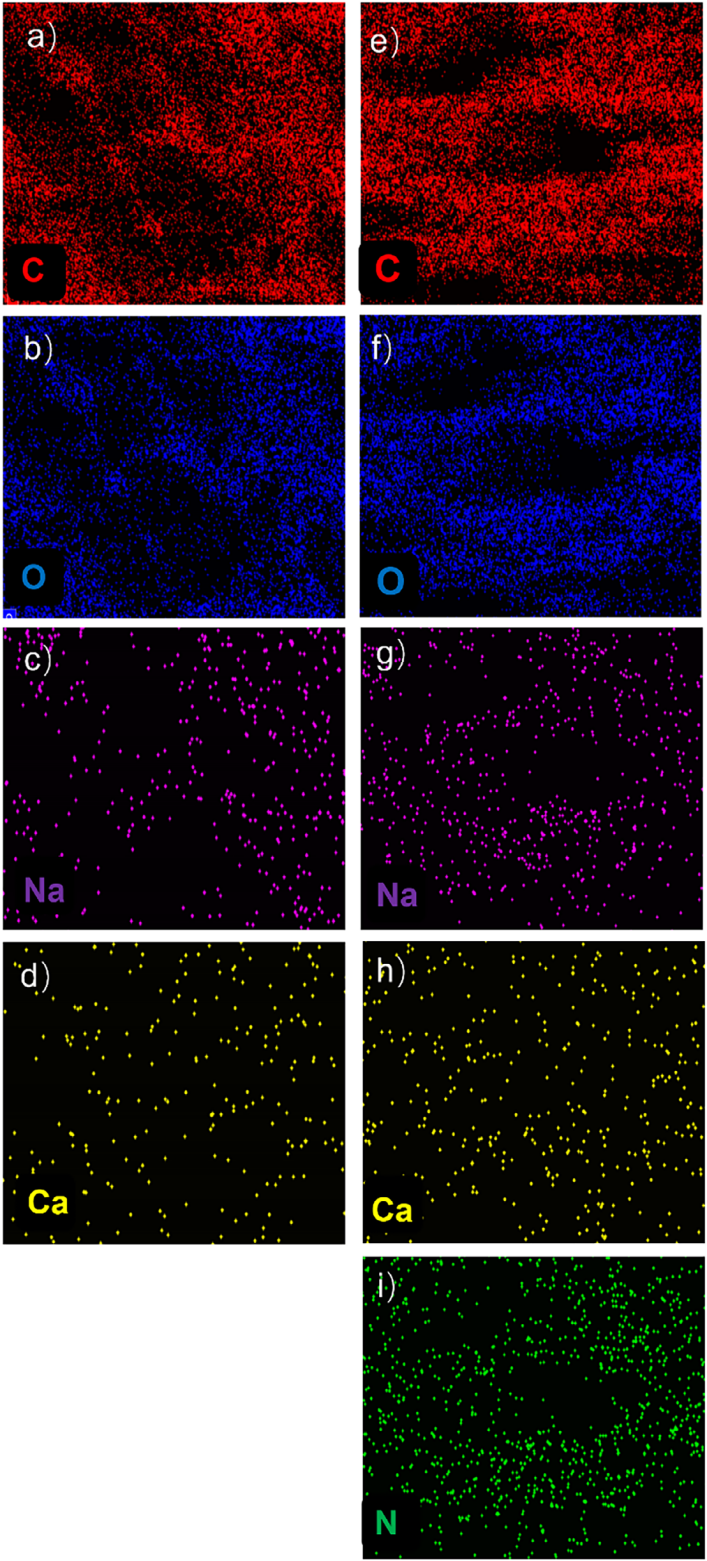
EDS mapping profiles of a–d) the SA hydrogels and e–i) the SA hydrogels embedded with SPN_2_/SA_1_ microgels.

To further address the successful introduction of the SPN_2_/SA_1_ microgels in the SA hydrogels, energy dispersive spectroscopy (EDS) mapping profiles are used to probe the elements on the cross‐section. Compared to the SA hydrogels without microgels (**Figure** **3**a–d), in addition, the element N can be observed in the SA hydrogels embedded with microgels (Figure 3–i). Since only SPN contains the element N, it can be concluded that the SPN/SA microgels are well installed in the SA hydrogels.

In addition, the morphology of microgels in the hybrid hydrogels is first characterized by optical microscopy. As shown in Figure S4 (Supporting Information), the SPN/SA microgels exhibit a sphere structure with uniform size dispersion. The sizes of eight microgels are measured as 427.5, 375, 443.8, 437.5, 406.3, 434.4, 381.3, and 418.8 µm. Thus, the average size of microgels is 405 ± 35 µm. To further analyze the distribution of SPN/SA microgels in the hybrid hydrogels, laser confocal scanning microscopy (LCSM) is applied as well. LCSM images (Figure S5, Supporting Information) clearly show the distribution of microgels in the hydrogel matrix. In addition, these microgels present a smooth surface and are homogenously dispersed throughout the hydrogel matrix.

### 2.3 Chemical Structure of Hybrid SA Hydrogels Embedded with SPN/SA Microgels

**Figure 4:**
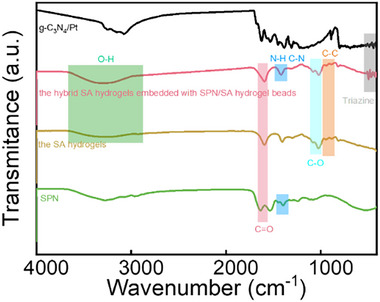
ATR‐FTIR spectra of SPN (green curve), g‐C_3_N_4_/Pt (black curve), the SA hydrogels (brown curve), and the hybrid SA hydrogels embedded with SPN_2_/SA_1_ microgels (pink curve).

The functional groups in SPN, g‐C_3_N_4_/Pt, the hybrid SA hydrogels, and the hybrid SA hydrogels embedded with SPN_2_/SA_1_ microgels are probed by ATR‐FTIR spectroscopy. In the spectrum of SPN (green curve in **Figure** **4**), the characteristic peaks at 1500–1700 cm^−1^ correspond to the amide I and amide II bands. In the spectrum of the hybrid SA hydrogels (brown curve in Figure 4), the characteristic absorption peaks located at 1000–1100 cm^−1^ are attributed to the stretching vibration of the C═O groups. In addition, an absorption peak corresponding to the C═C single bond is observed at 800–900 cm^−1^. Due to the introduction of the SPN/SA microgels, the hydrogen bonding is enhanced. Therefore, a prominent increase in the intensity of the broad absorption peaks of ─NH and ─OH groups is visible in the spectrum of hybrid SA hydrogels embedded with SPN_2_/SA_1_ microgels (red curve in Figure 4). In the spectrum of g‐C_3_N_4_/Pt nanosheets (black curve in Figure 4), the characteristic peak at 810 cm^−1^ corresponds to the tris‐s‐triazine unit. Besides that, several consecutive absorption peaks related to the C═N double bonds and C─N single bonds are visible in the range of 1200–1600 cm^−1^. A broad characteristic absorption peak at 3400–3700 cm^−1^ is observed as well, which is attributed to the stretching vibration of N─H groups. Although the amount of the g‐C_3_N_4_/Pt nanosheets is limited, the tri‐s‐triazine unit is still visible.

### 2.4 Light Absorption Capability

**Figure 5:**
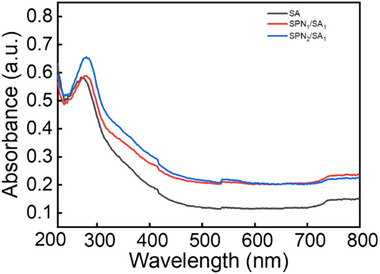
UV–vis spectra of the hybrid SA hydrogels (black curve), the hybrid SA hydrogels embedded with SPN_1_/SA_1_ microgels (red curve), and SPN_2_/SA_1_ microgels (blue curve).

The light absorbance capability of the hybrid SA hydrogels and the hybrid SA hydrogels embedded with SPN/SA microgels is determined by the UV–vis diffuse reflectance. Compared to the absorption capability of the hybrid SA hydrogels (black curve in **Figure** **5**), the hybrid SA hydrogels embedded with SPN_1_/SA_1_ microgels (mass ratio of SPN to SA = 1:1) present a much more pronounced absorption in the UV–vis light ranges (red curve in Figure 5). Moreover, the absorption is also related to the amount of SPN in the microgels. When the mass ratio of SPN to SA is increased from 1:1 to 2:1, a further increase of light absorption is observed in the hybrid SA hydrogels embedded with SPN_2_/SA_1_ microgels (blue curve in Figure 5). This is related to the different refractive indices of SPN and SA. According to the literature, SPN possesses a higher refractive index than that of SA.^[^ ^47^, ^48^ ^]^ Therefore, the refractive index of the SPN/SA microgels is also larger than that of the hybrid SA hydrogels. They can act as scattering centers for the incident light and significantly enhance the light‐harvesting capability. When the amount of SPN is increased in the microgels, the difference in the refractive indices turns larger. It favors the multi‐scattering of incident light in the hybrid SA hydrogels.

The number and size of microgels play an important role in light harvesting. When the microgel size is fixed at 400 µm and the number of microgels is increased from 10 to 100, the light absorption of the entire hybrid hydrogels exhibits a systematic and significant enhancement (Figure S6a, Supporting Information). This behavior is caused by the strengthening effect of the light scattering. Because each microgel acts as a light‐scattering center, adding more microgels to the hydrogels dramatically increases the possibility that incident light encounters the microgel surface. It further improves the light absorption in the hydrogel matrix, favoring the photocatalytic hydrogen evolution.

In addition, when the number of microgels is fixed as 50 and the size of microgels is increased from 100 to 800 µm, the total light absorption also presents an increasing tendency. The main reason is also related to the stronger scattering effect. Compared to the microgels with a small size (100 µm), there are more scattering points in the microgels with a large size (800 µm). Therefore, similar to the increase of the microgel number, the light scattering as well as the absorption are increased with the microgel size (Figure S6b, Supporting Information).

Due to the larger difference in the refractive index between SA and SPN, the pure SPN microgels embedded in the hybrid SA hydrogels should have a better light scattering capability than the SPN/SA microgels in the same hydrogel matrix. Therefore, the light‐harvesting capability should also be enhanced in the hybrid SA hydrogels embedded with pure SPN microgels. However, due to the lack of polymerization centers, the pure SPN microgels with a homogenous sphere structure cannot be prepared by the same protocol as the SPN/SA microgels. To avoid the possible influence of the different preparation protocols, only the hybrid SA hydrogels embedded with SPN/SA microgels are compared in the present investigation.

### 2.5 Water Retention Capability

**Figure 6:**
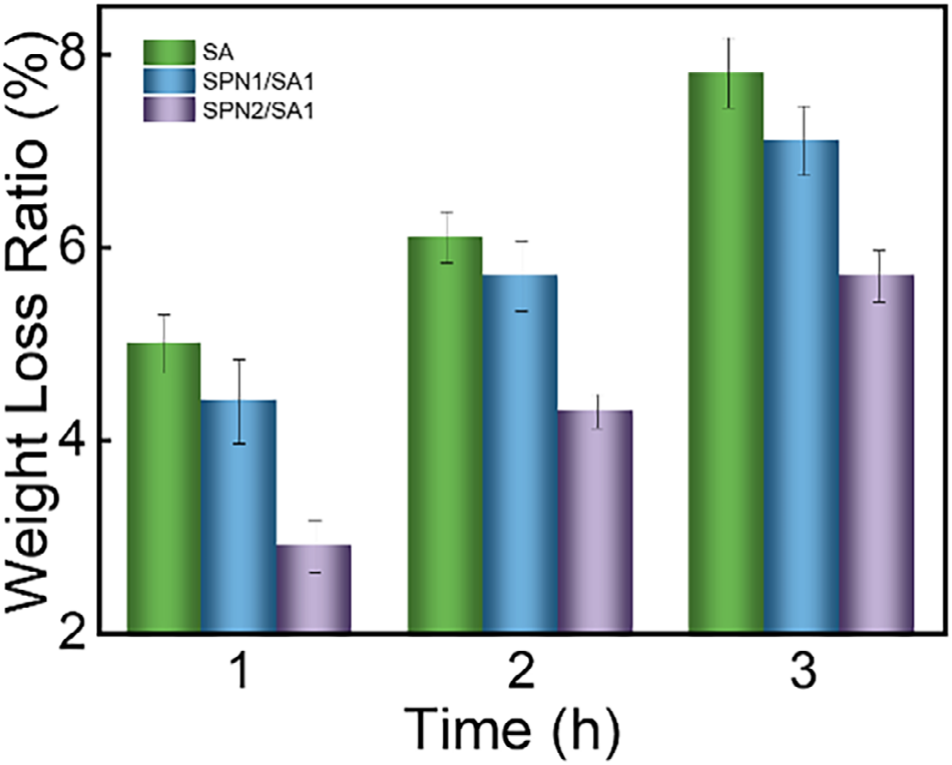
Weight loss of the hybrid SA hydrogels (green column), the hybrid SA hydrogels embedded with SPN_1_/SA_1_ microgels (blue column), and SPN_2_/SA_1_ microgels (purple column).

The water retention capability of the hybrid SA hydrogels embedded with SPN/SA microgels is studied via the temporal evolution of hydrogel weight under infrared illumination. As shown in the green column in **Figure** **6**, the hybrid SA hydrogels without microgels present the worst water retention capability. They lose 7.8% water after infrared illumination for 3 h. After the introduction of SPN_1_/SA_1_ microgels, the water retention capability is improved. The hybrid hydrogels embedded with SPN_1_/SA_1_ microgels (mass ratio of SPN to SA = 1:1, blue column in Figure 6) lose 7.1% in the same scenario. The water evaporation is only 90% of that of hybrid SA hydrogels without microgels. Further increasing the mass ratio of SPN to SA in the microgels to 2:1, the weight loss continues decreasing to 5.7% after infrared illumination for 3 h (purple column in Figure 6). As a protein, SPN is extremely hydrophilic. It can absorb and bond with a large number of water molecules. The larger amount of protein indicates more hydrophilic groups, which favors the presence of water molecules in hydrogels. In addition, the electrostatic interaction and hydrogen bonding between SPN also stabilize the water molecules in the hydrogels. Combining these two factors, the water retention capability is profoundly improved in the hybrid SA hydrogels embedded with SPN/SA microgels.

As shown in Figure 6, the weight loss is reduced with the amount of SPN in SPN/SA microgels. Thus, it can be concluded that the pure SPN microgels should have a better water retention capability than the SPN/SA microgels. However, as mentioned above, the pure SPN microgels cannot be prepared by the same protocol as the SPN/SA microgels and are therefore not part of the present study.

### 2.6 Photocatalytic Hydrogen Evolution

**Figure 7:**
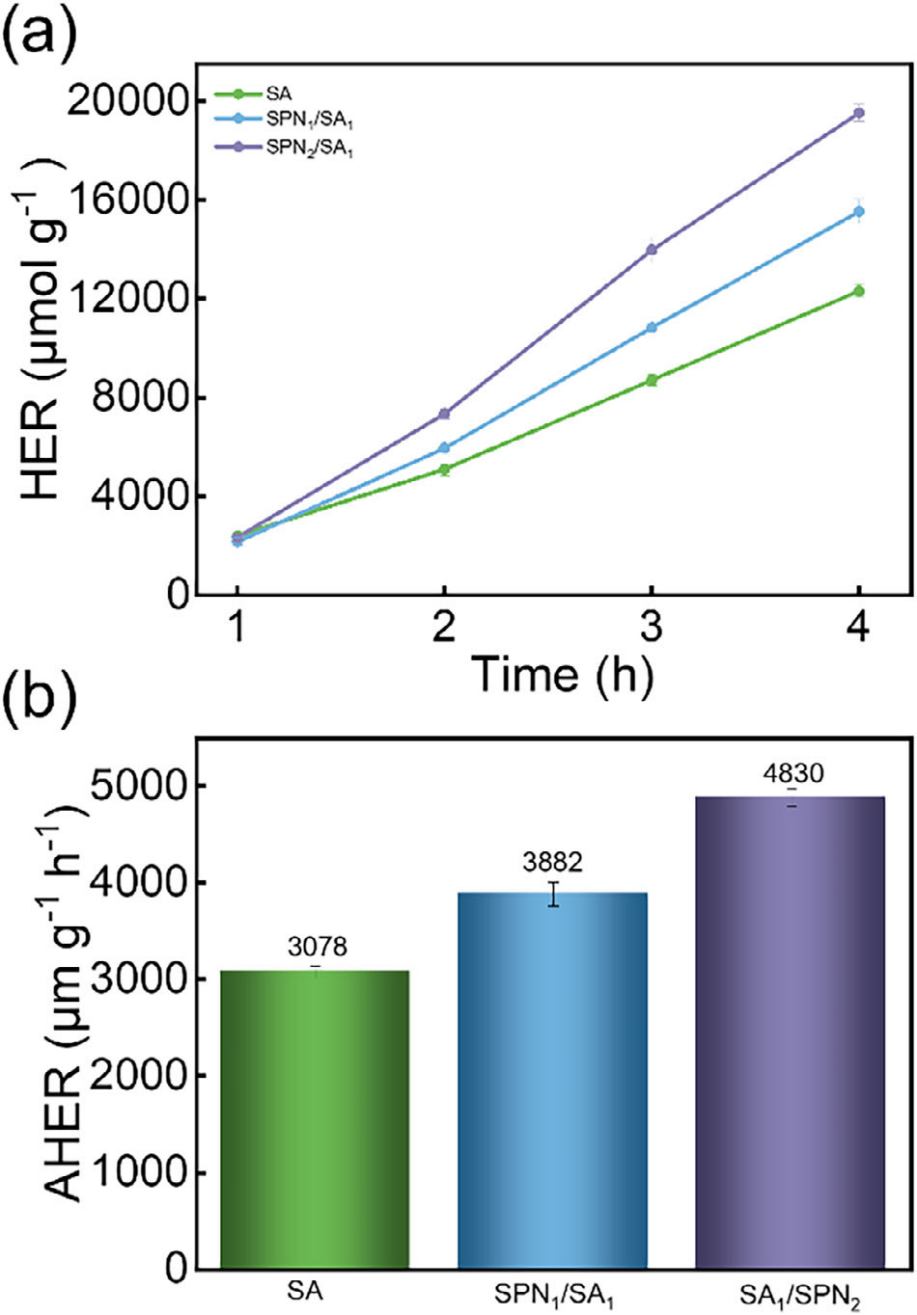
a) Hydrogen evolution of the hybrid SA hydrogels (green curve), the hybrid SA hydrogels embedded with SPN_1_/SA_1_ microgels (blue curve), and SPN_2_/SA_1_ microgels (purple curve). b) Average HER values in the hybrid SA hydrogels (green column), the hybrid SA hydrogels embedded with SPN_1_/SA_1_ microgels (blue column), and SPN_2_/SA_1_ microgels (purple column).

The photocatalytic hydrogen evolution in the hybrid SA hydrogels, as well as the hybrid SA hydrogels embedded with SPN/SA microgels, is measured by gas chromatography. After being illuminated by a xenon lamp for 1 h, the hydrogen evolution of the hybrid SA hydrogels is 2407 µmol g^−1^ (green curve in **Figure** **7**). Further prolonging the illumination time, the hydrogen evolution shows a linear increase with time. After exposure for 4 h, the final hydrogen evolution can reach 12 313 µmol g^−1^. Thus, the average hydrogen evolution rate (HER) value for the hybrid SA hydrogels is 3078 µmol g^−1^ h^−1^ (green column in Figure 7). When the SPN/SA microgels (mass ratio of SPN to SA = 1:1) are introduced, the hybrid SA hydrogels embedded with SPN_1_/SA_1_ microgels present an enhanced photocatalytic performance. The hydrogen evolution is 2173 µmol g^−1^ (blue curve in Figure 7) in the first hour. This value is slightly smaller than that of the hybrid SA hydrogels without microgels. This difference might be related to the difficulty of hydrogen diffusion in the presence of embedded microgels. However, when the time is prolonged, the linear increase of hydrogen evolution is more pronounced. The final hydrogen evolution reaches 15 529 µmol g^−1^ after 4 h. Thus, the average HER value rises to 3 882 µmol g^−1^ h^−1^, which is 26% larger than that of the hybrid SA hydrogels. Further increasing the mass ratio of SPN to SA to 2:1, both the hydrogen evolution and the average HER of the hybrid SA hydrogels embedded with SPN_2_/SA_1_ microgels continue increasing (purple curve and column in Figure 7). The average HER value is 4830 µmol g^−1^ h^−1^, which is 157% of that of hybrid SA hydrogels. Therefore, it can be concluded that the introduction of SPN/SA microgels into the hybrid SA hydrogels indeed favors the photocatalytic water splitting. Due to the different refractive indices of SPN/SA microgels and hybrid SA hydrogels, the microgels can scatter the incident light in the hydrogels. Compared to the direct penetration of the incident light in the semi‐transparent hybrid SA hydrogels, the multi‐scattering of incident light improves the light harvesting capability and enhances the photocatalytic hydrogen evolution. Moreover, the good water retention also slows down the water evaporation rate of the hybrid SA hydrogels, which is favorable for the hydrogen evolution as well.

**Figure 8:**
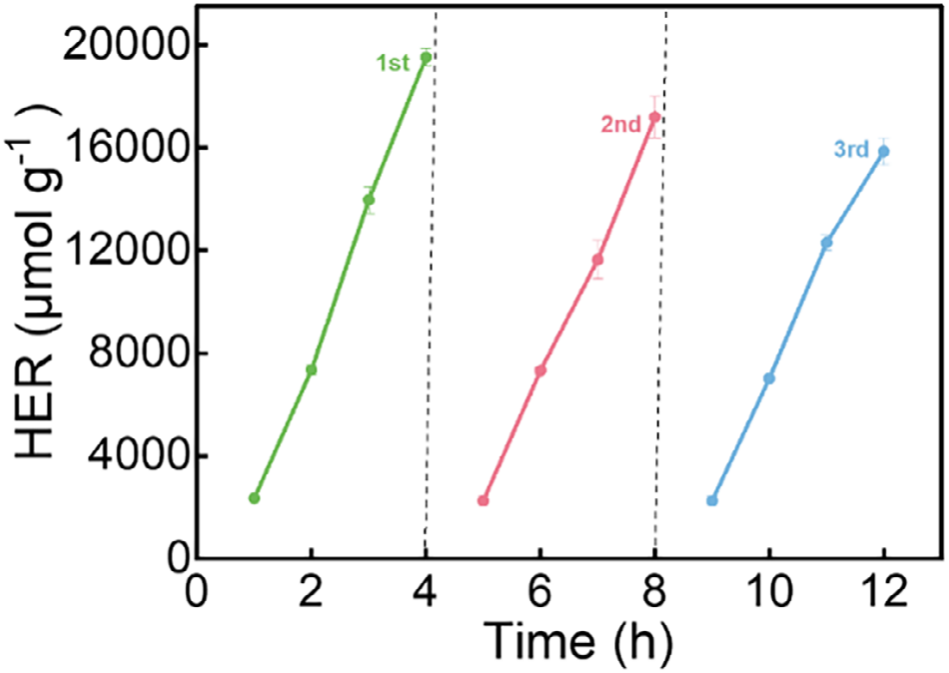
HER during the cyclical measurements of the hybrid SA hydrogels embedded with SPN_2_/SA_1_ microgels.

In addition, due to less evaporation in the hybrid SA hydrogels embedded with SPN/SA microgels, the structural deformation caused by the evaporation is much less prominent. Thus, even after three cycles of hydrogen evolution and refilling with water/sacrificial agent, the hybrid SA hydrogels embedded with SPN/SA microgels still present a good photocatalytic performance (**Figure** **8**).

To compare the HER of the present hybrid hydrogel system embedded with SPN/SA microgels with other photocatalytic systems using hydrogel matrices, the average HER values are taken from the literature. The selected systems are hybrid hydrogels containing g‐C_3_N_4_/Pt nanosheets,^[^ ^14^ ^]^ hybrid hydrogels containing g‐C_3_N_4_/Pt nanosheets covered with PVDF membrane,^[^ ^26^ ^]^ and hybrid microgels embedded with g‐C_3_N_4_/Pt nanosheets,^[^ ^25^ ^]^ and the average HER (AHER) values are listed in Table S1 (Supporting Information). Compared to the hybrid hydrogels and hybrid hydrogels covered with PVDF membrane, the additional SPN/SA microgels embedded in the hybrid hydrogel system significantly enhance the AHER. This behavior is related to the better light harvesting caused by the scattering and good water retention by the additional hydrogen bonding. The hybrid microgels show a similar AHER as the hybrid hydrogels embedded with SPN/SA microgels, which can be attributed to the larger specific surface area of hybrid microgels.

## 3 Conclusion

Efficient hydrogen evolution and water retention are realized in the hybrid SA hydrogels containing g‐C_3_N_4_/Pt nanosheets by introducing the SPN/SA microgels. Due to the refractive index of SPN being larger than that of SA, the SPN/SA microgels can act as the scattering centers in the hybrid SA hydrogels. Thus, the incident light is efficiently scattered, and the light‐harvesting capability is improved, favoring the photocatalytic water splitting via the g‐C_3_N_4_/Pt nanosheets. When the mass ratio of SPN to SA is 2:1, the average HER value of the hybrid SA hydrogels embedded with SPN_2_/SA_1_ microgels can reach 4830 µmol g^−1^ h^−1^, which is 57% larger than that of the hybrid SA hydrogels without any microgels. In addition, the existence of ─COOH and ─NH_2_ groups in SPN enhances the electrostatic interaction and hydrogen bonding with water molecules. Thereby, the ability to keep water molecules in the hybrid SA hydrogels is also improved. Compared to the hybrid SA hydrogels without microgels, the evaporation rate is 27% slower. The reduced evaporation and better light harvesting indicate that the present hybrid SA hydrogels embedded with SPN/SA microgels can perform photocatalytic water splitting with high efficiency for a long time. They are very suitable for the hydrogen production in areas rich in sunlight but poor in water, such as prairies and deserts.

## Conflict of Interest

The authors declare no conflict of interest.

## Supporting information



Supporting Information

## Data Availability

The data that support the findings of this study are available from the corresponding author upon reasonable request.
