# Supplementary material for: Efficient Light Harvesting and Water Retention Realized by Soybean Protein‐Based Microgels Embedded in Hybrid Sodium Alginate Hydrogels Containing Photocatalyst for Hydrogen Evolution
Source: Adv Sci (Weinh). 2025 Nov 7;13(6):e05118. doi: 10.1002/advs.202505118 (PMC12866813; doi:10.1002/advs.202505118)
Supplement: Supplementary file 1 — Supporting Information [file ADVS-13-e05118-s001.pdf]

Supporting Information

Efficient Light Harvesting and Water Retention Realized by Soybean Protein-based Microgels Embedded in Hybrid Sodium Alginate Hydrogels Containing Photocatalyst for Hydrogen Evolution

Jie Yu,[1] Neng Hu,[1] Weijia Wang,[2,] Lin Lei,[2] Huiqing Fan,[2] Peter Müller-Buschbaum[3,*] and Qi Zhong[1,3,*]*

Experimental Section

1. Materials

Sodium alginate (SA, AR, 90%), soybean protein isolate (SPI) and sodium hydroxide (NaOH, AR, 96%) were purchased from Shanghai Macklin Biochemical Co., Ltd. Calcium chloride ($CaCl_2$) was purchased from Hangzhou Gaojing Fine Chemical Industry Co., Ltd. Hydrochloric acid was purchased from Huzhou Shuang Chemical Technology Co., Ltd. Dicyandiamide ($C_2H_4N_4$, AR) was bought from Aladdin.

2. Preparation of g-C_3N_4/Pt Nanosheets

The preparation of the g-C_3N_4 nanosheets were as follows: Dicyandiamide was calcined in air at 550 °C with a temperature increase rate of 5 °C min^{-1} for 4 h to obtain lumpy g-C_3N_4, which was further ground into powder by an agate mortar. Then, the g-C_3N_4 powder was calcined at 550 °C for 2 h at a heating rate of 2 °C min^{-1}. The obtained g-C_3N_4 nanosheets were yellow powder.

The loading of Pt atoms onto the g-C_3N_4 nanosheets was realized by the $NaBH_4$ reduction strategy. In general, the g-C_3N_4 nanosheets (200 mg) were added into the deionized water with rotation. Then, H_2PtCl_6 solution (1 mL, 2 mg mL^{-1}) and $NaBH_4$ (400 mg) were sequentially added into the suspension and continue rotating for 30 min. The suspension was filtered and sequentially washed with deionized water and ethanol. After drying in an oven thermo-stated at 80 °C for 12 h, the g-C_3N_4 nanosheets loaded with Pt atoms were obtained.

3. Preparation of soybean protein nanofibers (SPN)

Soybean protein isolate (SPI, 50 mg L^{-1}) was uniformly distributed in deionized water and the pH value was adjusted to 2.0. After rotating the solution for 2 h at room temperature, the SPI was fully dissolved. The solution was stored overnight at 4 °C to realize the full hydration.

Subsequently, the hydrated solution was centrifuged at 10,000 rpm for 30 min. The obtained supernatant was then filtered via a 0.22 μm filter membrane to remove the undissolved large particles. Then the solution was heated at 85 °C for 12 h with continuous rotation. Finally, the protein solution was quenched in ice water and adjusted the pH value to 7.0. The solution was freeze-dried to obtain soybean protein nanofibers (SPN).

4. Preparation of SPN/SA microgels

The preparation of the SPN/SA microgels was as follows. The SA solution (1 wt%, 10 mL) and SPN solution (1 wt%, 10 mL) were mixed at room temperature, and rotated at 600 pm for 4 h to ensure the complete mixture. The mass ratio of SPN to SA was 1:1. The mixed solution was dropped into a $CaCl_2$ solution (1 wt%, 100 mL) via a syringe. To avoid the aggregation of the microgels, the $CaCl_2$ solution was rotated at 300 rpm. After maintaining the $CaCl_2$ solution for 6 h, the gelation process of SPN/SA microgels was completed. The obtained SPN/SA microgels were rinsed with deionized water three times to remove the possible impurities on the surface and named SPN_1/SA_1 microgels. The diameter for the SPN_1/SA_1 microgels was around 400 μm (Figure S4).

To address the influence of SPN on light harvesting and water retention, the mass ratio of SPN to SA was changed to 2:1 and named SPN_2/SA_1 microgels. The preparation protocol for the microgels was identical.

5. Preparation of hybrid SA hydrogels embedded with SPN/SA microgels

After treatment with ultrasound for 15 min, the g-C_3N_4/Pt nanosheets (2 mg) were uniformly dispersed in the SA solution (1 wt%, 10 mL). Subsequently, 100 SPN/SA microgels were added into the dispersion with rotation. Then, the hybrid SA hydrogels embedded with SPN/SA microgels were cross-linked by immersing in a $CaCl_2$ solution (1 wt%, 50 mL) for 24 h. Finally, the obtained hybrid SA hydrogels embedded with microgels were moved in deionized water for 12 h to remove any residual impurities.

6. Microscopy measurements

The structure of SPN was probed via transmission electron microscope (JEM-1400Flash, JEOL, Japan). The SPN solution was first diluted with deionized water and dropped on a 200-mesh carbon-coated copper grid. After drying at 25 °C for 90 min, the samples were coated with tungsten phosphate. The voltage applied was 120 kV.

Field-emission SEM (ULTRA55, Carl Zeiss SMT Pte. Ltd., Germany) was used to probe the cross-section morphology of the hybrid SA hydrogels and the hybrid SA hydrogels embedded with SPN/SA microgels. The corresponding operating voltages and distance were set as 8 kV and 10 mm, respectively. Before the measurements, these hydrogels were first dehydrated by a freeze dryer (Lab-1A-50, BIOCOOL, China) and then sputtered with platinum for 110 s by an automated fine coater (JFC-1600, JEOL, Japan).

7. Thioflavin T fluorescence of SPN (ThT)

The SPN was first dissolved in phosphate buffer solution (PBS) with a pH value of 7.0. The samples were mixed with the ThT working solution and incubated for 20 min at room temperature to allow ThT fiber binding. The intensity was measured by a fluorescence spectrometer with an excitation wavelength of 450 nm and an emission wavelength of 482 nm.

8. Characterization of functional groups

The functional groups in the hybrid SA hydrogels and the hybrid SA hydrogels embedded with SPN/SA microgels were measured by the Fourier-transform infrared spectroscopy with an ATR module on a Vertex 70 spectrometer (ATR-FTIR, Bruker, USA). The wavenumber was from 600 to 4000 cm^{-1}. The resolution and scan number were 4 cm^{-1} and 32, respectively.

9. UV-vis absorption spectra measurements

The UV-vis absorbance of the hybrid SA hydrogels embedded with SPN/SA microgels was investigated by a UV-vis spectrophotometer with an integrated sphere (UV-2600, PerkinElmer, USA). The wavelength range was selected as 200-800 nm.

10. Water retention capability measurements

The water retention capability was investigated by temporal weight loss of hybrid SA hydrogels embedded with SPN/SA hydrogel beads under infrared illumination. An IR lamp (Philips, Korea) was used as the light source. The light intensity of the IR lamp was measured by a laser power meter (LP-3B, Beijing Wuke Photoelectric Technology Co., Ltd.). When the distance between the IR lamp and the sample was 10 cm, the light intensity was 3079.6Wm^{-2}. Before the measurements, the weight of the as-prepared the hybrid SA hydrogels embedded with SPN/SA hydrogel beads was first measured. After illumination for 1 and 2 h, the weight was measured again. To address the influence of the SPN/SA microgels to the water retention

capability, the hybrid SA hydrogels without the SPN/SA microgels were also measured with the identical protocol.

11. Photocatalytic hydrogen evolution measurements

A xenon lamp (HDL-II, Bobei Lighting Electrical Factory, China) was used as the light source for the photocatalytic water splitting. A laser power meter (LP-3B, Beijing Wuke Optoelectronics Technology Co., Ltd, China) was used to measure the light intensity of the xenon lamp. When the distance between the lamp and the hybrid SA hydrogels was 10 cm, the light intensity was 573.25 W m^{-2}. No filter was applied in the light source. Since the hybrid SA hydrogels and the hybrid SA hydrogels embedded with SPN/SA microgels were both measured with the same light source, the influence of the light source can be neglected. The sacrificial agent used in the photocatalytic hydrogen evolution was triethanolamine (TEOA). Before the measurements, the hybrid hydrogels were immersed in a mixed solution containing TEOA (4 mL) and distilled water (10 mL) for 48 h.

Before the photocatalytic water splitting measurements, N_2 was injected into the sealed glass tube to establish an N_2 atmosphere. After every interval (1 h), 400 μL of gas was withdrawn from the sealed glass tube by a syringe (1 mL). The photocatalytic hydrogen production was measured by a TCD gas chromatograph (GC1690, Kulun Technology Co., Ltd, China).

12. Statistical analysis

The data in this manuscript are primarily presented in the form of bar charts and line graphs. For the total hydrogen evolution and hydrogen evolution cycle diagrams, the data are averaged, and the error bars are obtained from the standard deviation processing to address the reliability of the data. All data processing was performed by the software Origin.

16. Calculation of the Scattering Coefficient

A reliable and more straightforward calculation is applied in our present investigation to obtain the scattering coefficient (equation 1):

$$\mu s = Qs\,A\,\rho \qquad (1)$$

in which, Q_S is the total scattering efficiency of the microgels. In our present investigation, the value is around 2 for particles. A is the cross-sectional area of microgels, and ρ is the concentration of microgels. The volume of the hybrid hydrogels is 0.4 cm^3. The diameter of microgels is 405 μm. There are 50 microgels embedded in the hybrid hydrogels. Therefore, μ_s can be calculated as 0.163 cm^{-1}.

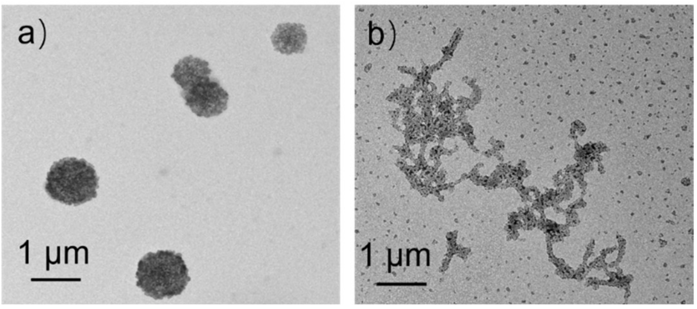

Figure S1. TEM images of (a) SPI and (b) SPN.

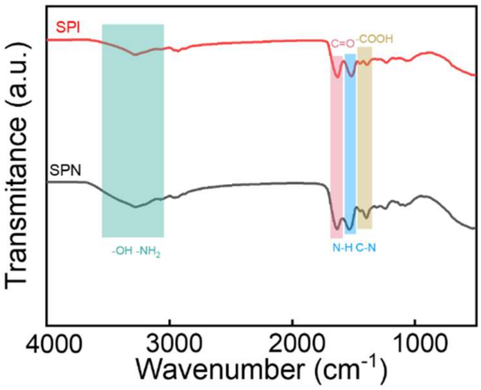

Figure S2. ATR-FTIR spectra of SPI and SPN.

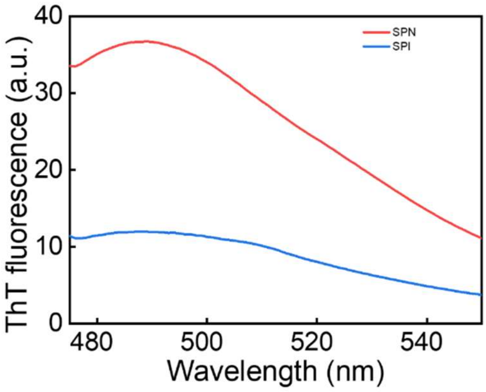

Figure S3. ThT binding fluorescence intensity of SPI and SPN.

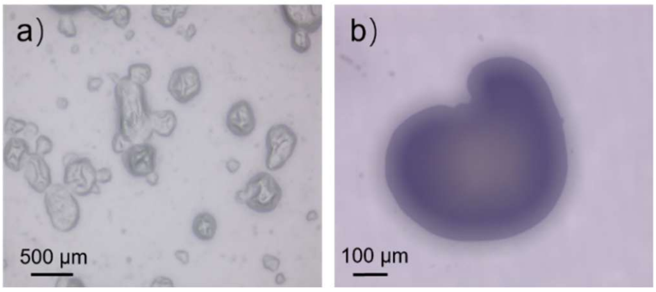

Figure S4. Optical microscope images of the SPN/SA microgels.

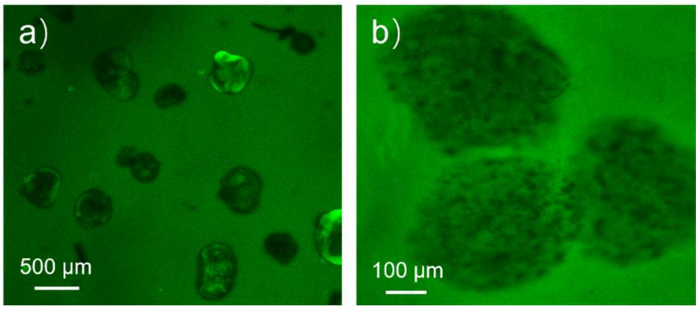

Figure S5. LCSM images of the SPN/SA microgels in the hybrid hydrogels.

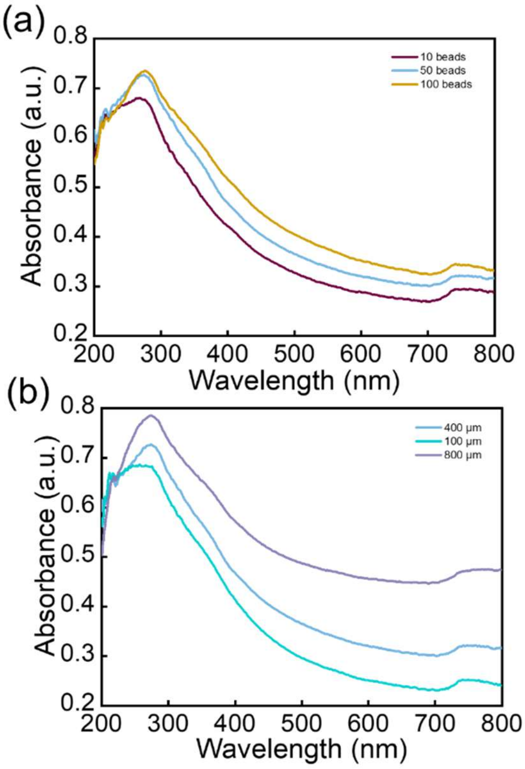

Figure S6. UV-vis spectra of the hydrogels embedded with different (a) number and (b) size of SPN/SA microgels.

Table S1. Comparison of different photocatalytic hydrogen production systems.

	hybrid hydrogels	PVDF Membrane on hybrid hydrogels	hybrid microgels
AHER ($\mu mol\ g^{-1}\ h^{-1}$)	1842	2543	4994
